# Analysis of Total Thiols in the Urine of a Cystathionine β-Synthase-Deficient Mouse Model of Homocystinuria Using Hydrophilic Interaction Chromatography

**DOI:** 10.3390/molecules25071735

**Published:** 2020-04-09

**Authors:** Chun-Fang Chang, Kenji Hamase, Makoto Tsunoda

**Affiliations:** 1 Graduate School of Pharmaceutical Sciences, The University of Tokyo, Tokyo 1130033, Japan; chunfang31@gmail.com; 2 Graduate School of Pharmaceutical Sciences, Kyushu University, Fukuoka 8128582, Japan; hamase@phar.kyushu-u.ac.jp

**Keywords:** high-performance liquid chromatography, ammonium 7-fluoro-2,1,3-benzoxadiazole-4-sulfonate, tris(2-carboxyethyl) phosphine, homocysteine

## Abstract

Homocysteine and related thiols (cysteine, cysteinylglycine, and glutathione) in the urine of a cystathionine β-synthase (CBS)-deficient mouse model were quantified using hydrophilic interaction chromatography with fluorescence detection. Urine samples were incubated with tris(2-carboxyethyl) phosphine to reduce disulfide bonds into thiols. After deproteinization, thiols were fluorescently derivatized with ammonium 7-fluoro-2,1,3-benzoxadiazole-4-sulfonate (SBD-F). Homocysteine, cysteine, cysteinylglycine, and glutathione in mouse urine were analyzed using an amide-type column with a mobile phase of acetonitrile/120 mM ammonium formate buffer (pH 3.0) (81:19). The developed method was well-validated. Thiol concentrations in the urine of CBS-wild type (-WT), -heterozygous (-Hetero), and -knockout (-KO) mice were quantified using the developed method. As expected, total homocysteine concentration in CBS-KO mice was significantly higher than that in CBS-WT and CBS-Hetero mice. The developed method shows promise for diagnoses in preclinical and clinical studies.

## 1. Introduction

**Figure 1:**
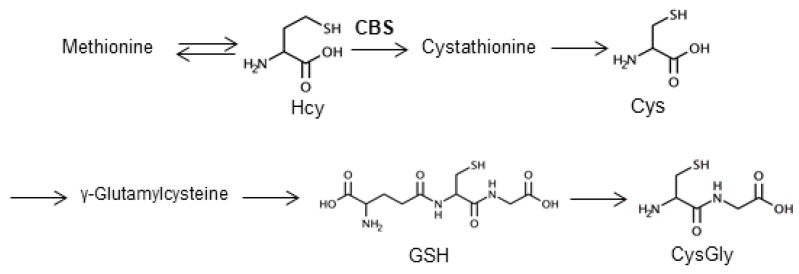
Metabolism pathway of homocysteine-related thiols and structures of target thiol compounds. Hcy, homocysteine; Cys, cysteine; GSH, glutathione, CysGly, cysteinylglycine, CBS, cystathionine β-synthase.

Homocystinuria is an inherited disease caused by a cystathionine β-synthase (CBS) deficiency. CBS converts homocysteine (Hcy) into cystathionine. When CBS is impaired, Hcy might accumulate in blood and urine, leading to severe symptoms such as ectopia lentis, learning difficulties, and skeletal abnormalities [1]. Other defects such as re-methylation defects (methionine synthase deficiency) and vitamin deficiencies also lead to homocystinuria. As homocystinuria produces markedly increased Hcy concentrations in plasma and urine, analysis of the total Hcy is used to diagnose and monitor disease progression [2]. Hcy has several metabolites—it can be remethylated to methionine or converted to cysteine (Cys). Glutathione (GSH) is synthesized from Cys and glutamate. Cysteinylglycine (CysGly) is generated from degradation of GSH (Figure 1). In addition to measuring Hcy concentration, determining the balance between Hcy and its metabolites (Cys, GSH, and CysGly) might help elucidate the pathology of homocystinuria with CBS deficiency. As urine is less hazardous and can be collected by noninvasive means [3], it is preferred over blood specimens. However, analysis of urinary thiols is hampered by the presence of other endogenous compounds, thus, urine is seldom analyzed for thiols [4].

In clinical studies, total thiols, the sum of reduced and oxidized (symmetrical disulfides, mixed disulfides, and protein-bound thiols) forms are generally determined. The most common method for determination of thiols is high-performance liquid chromatography (HPLC) [5]. In our previous studies [6,7], we utilized hydrophilic interaction chromatography (HILIC) to separate thiols in biological fluids, including human plasma and mouse serum. Pretreatment was followed by reduction with tris(2-carboxyethyl) phosphine (TCEP), deproteinization, and derivatization with ammonium 7-fluoro-2,1,3-benzoxadiazole-4-sulfonate (SBD-F). Since HILIC can separate highly polar compounds such as SBD-thiols [8,9], HILIC with fluorescence detection was developed to analyze the total homocysteine and its metabolite concentrations, in the plasma of CBS-wild type (CBS+/+, -WT), -heterozygous (CBS+/−, -Hetero), and -knockout (CBS−/−, -KO) mice [10].

As urine samples can also be useful for clinical diagnosis, the urine of CBS-WT, -hetero, and -KO mice was analyzed for thiol compounds. After optimizing the conditions for HILIC separation with an amide-type column, thiols in the urine of CBS-WT, -hetero, and -KO mice were quantified. Hcy and related thiol concentrations were investigated as a means of homocystinuria evaluation.

## 2. Results and Discussion

### 2.1. Optimization of Separation Conditions Using an Amide-Type Column for SBD-Thiols in Urine

**Figure 2:**
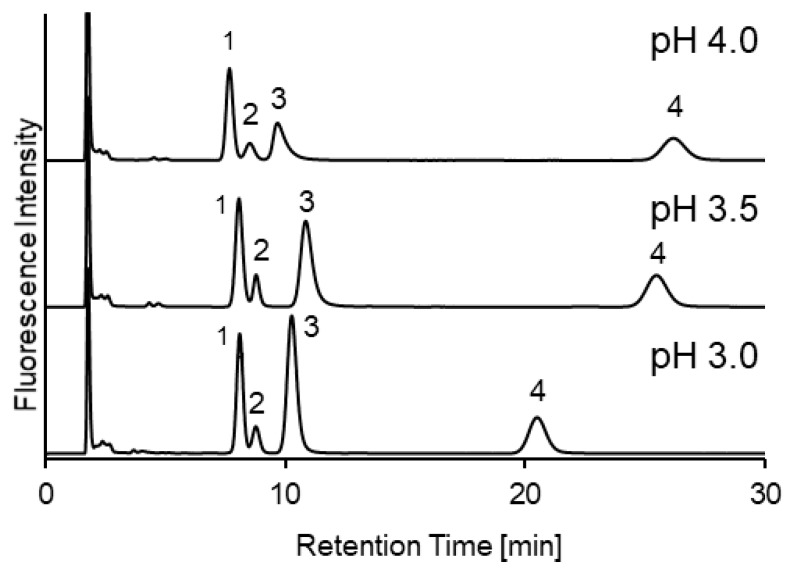
Chromatograms of ammonium 7-fluoro-2,1,3-benzoxadiazole-4-sulfonate (SBD)-thiol standard solution under different buffer pH. Mobile phase: acetonitrile/40 mM ammonium formate (80:20). Peaks—1, SBD-homocysteine (-Hcy); 2, SBD- cystathionine (-Cys); 3, SBD-Cysteinylglycine (-CysGly); and 4, SBD-Glutathione (-GSH).

We previously developed an HILIC method for thiols in human plasma and mouse sera, using the precolumn fluorescence derivatization reagent, SBD-F [6,7]. In this study, the same mobile phase (acetonitrile/40 mM ammonium formate buffer (pH 3.0) (80:20)) was applied for urine analysis. However, a poor separation was observed, since urine exhibited more endogenous peaks than plasma. Hence, the suitability of the mobile phase was re-examined. We first investigated the effect of buffer pH on SBD-thiols retention. As shown in Figure 2, with increasing buffer pH, the SBD-Hcy and -Cys resolution improved; however, analysis time also increased. Furthermore, SBD-CysGly peak tailing occurred with increasing pH. As buffer pH does not affect the stationary phases, the poor peak shape might have been caused by a change in the charge(s) of the analytes (pKa of CysGly is 3.6). Considering analysis time and peak symmetry a buffer pH 3.0 was selected.

**Figure 3:**
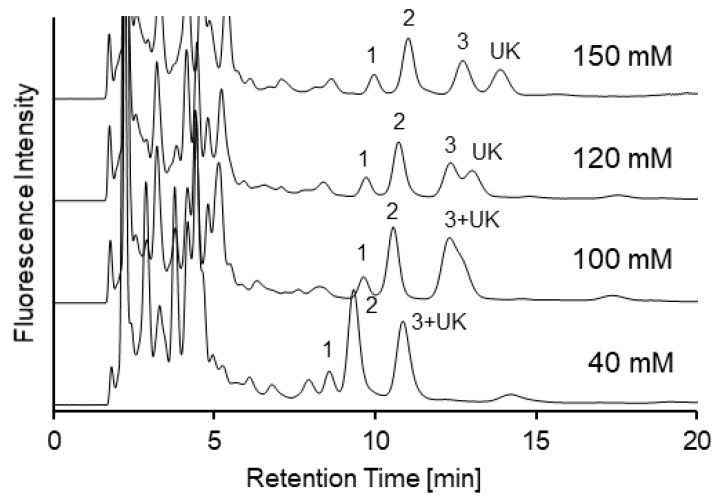
Chromatograms of mouse urine samples with different buffer concentration. Mobile phase—acetonitrile/ammonium formate (pH 3.0) (80:20). Peaks—1, SBD-Hcy; 2, SBD-Cys; 3, SBD-CysGly; UK, unknown peak.

Next, for improved separation, ammonium formate buffer concentration was investigated using urine samples. Resolution between SBD-Hcy and -Cys was improved at higher concentrations (Figure 3). When the concentration was higher than 100 mM, baseline separation (resolution, Rs > 1.5) between SBD-Hcy and -Cys was achieved. However, an unknown peak in mouse urine samples overlapped with the SBD-CysGly peak. Buffer concentration was increased to 150 mM, and near baseline separation between SBD-CysGlys and the unknown peak was achieved. However, high concentrations of ammonium formate are not soluble at high organic solvent content [11]. Hence, 120 mM ammonium formate buffer was chosen as the optimal concentration.

**Figure 4:**
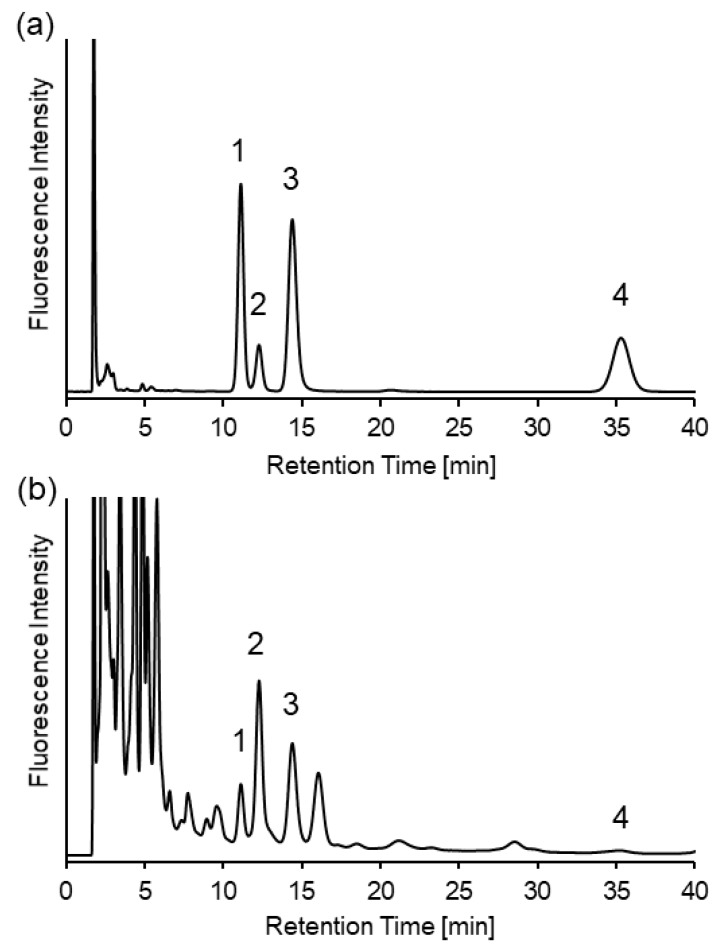
Chromatograms of (**a**) 1 µM SBD-thiols standard solution and (**b**) mouse urine samples under optimized conditions. Mobile phase—acetonitrile/120 mM ammonium formate (pH 3.0) (81:19). Peaks—1, SBD-Hcy; 2, SBD-Cys; 3, SBD-CysGly; 4, SBD-GSH.

For the baseline separation between the SBD-CysGly and the unknown peaks, acetonitrile content was further increased. With a mobile phase of acetonitrile/120 mM ammonium formate (pH 3.0) (81:19), four thiol compounds (Hcy, Cys, CysGly, and GSH) were separated within 40 min. Resolution above 1.5 was reached for all thiols and unknown peaks. Chromatograms of the standards and mouse urine under optimal conditions are shown in Figure 4.

### 2.2. Method Validation

Linearity, sensitivity, precision, and accuracy of the developed method were validated. Table 1 shows the linear range, limits of detection (LOD), and limits of quantitation (LOQ). The calibration curves for each thiol compound showed good linearity (R^2^ > 0.999). The LODs for SBD-thiols were about 2–143 times lower than those previously reported using reversed-phase liquid chromatography [12,13,14]. This might result from the use of an acetonitrile-rich mobile phase in HILIC, which enhanced the fluorescence intensity of SBD-thiols and improved sensitivity [7,15].

As shown in Table 2, the intra-day precision was less than 3.8%, and the recovery range was 93% to 119% for the mouse urine samples. The inter-day precision was below 7.3%, and the recovery range was 87% to 110% (Table 3). These results validated the developed method.

### 2.3. Analysis of Biothiols in Urine Samples of Mice with Cystathionine β-Synthase Deficiency

**Figure 5:**
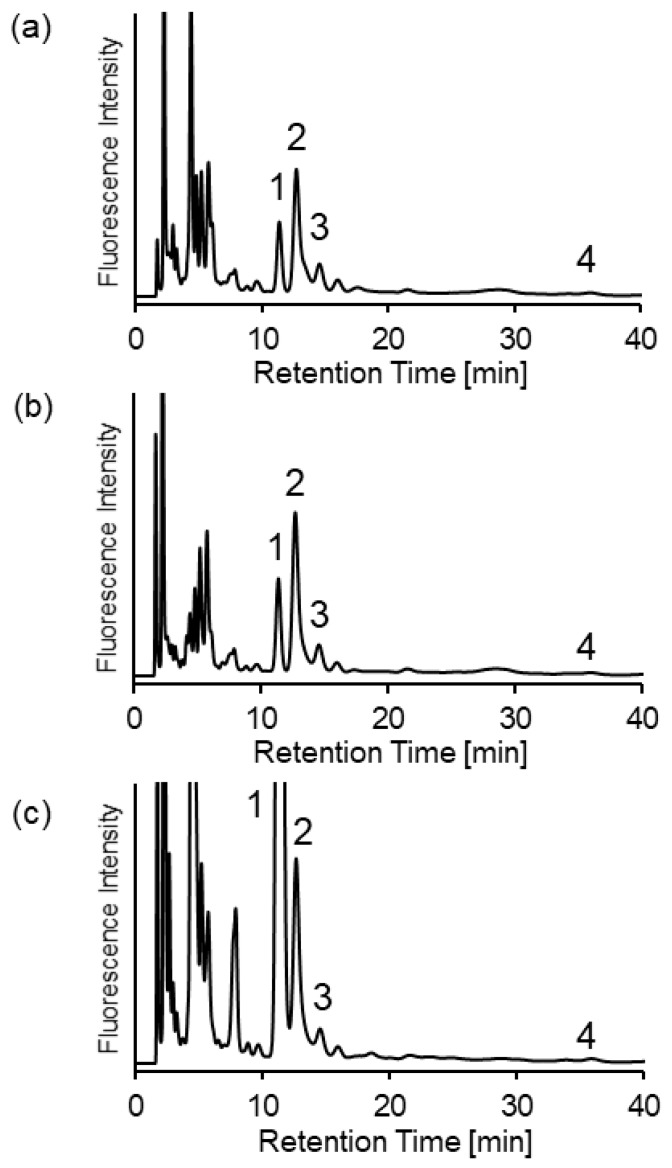
Chromatograms of (**a**) CBS-wild type (CBS-WT), (**b**) CBS-heterozygous (CBS-Hetero), and (**c**) CBS-knockout (CBS-KO) mouse urine samples. Mobile phase—acetonitrile/120 mM ammonium formate (pH 3.0) (81:19). Peaks—1, SBD-Hcy; 2, SBD-Cys; 3, SBD-CysGly; 4, SBD-GSH.

Urine samples from CBS-WT, -Hetero, and -KO mice were analyzed using the established method. Four SBD-thiol compounds (Hcy, Cys, CysGly, and GSH) in urine from each type of mouse were separated, as shown in Figure 5.

**Figure 6:**
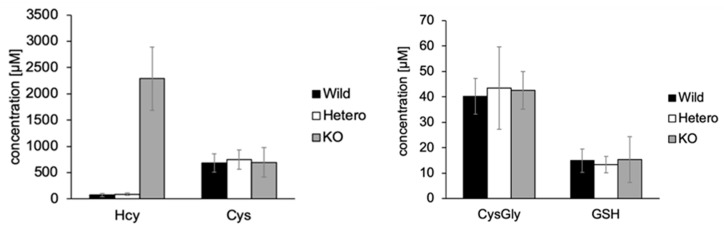
Concentration of thiols in CBS-WT, -Hetero, and -KO mouse urine samples. Numbers of samples—WT, *n* = 4; Hetero, *n* = 9; KO, *n* = 7. Error bars—standard deviation.

Thiol concentrations in mouse urine samples were calculated from calibration curves (described in Section 2.2) (Figure 6). Excretions of total Hcy in the urine of CBS-WT, -Hetero, and -KO mice were 73.6 ± 33.7 µM (*n* = 4), 89.2 ± 21.8 µM (*n* = 9), and 2288.7 ± 600.5 µM (*n* = 7) (mean ± SD), respectively. As expected, total Hcy in CBS-KO mouse urine samples was extremely elevated, compared to those measured in CBS-WT and CBS-Hetero. The urinary excretion of total Hcy in CBS-Hetero and CBS-KO were approximately in agreement with previous studies [16]. Although the total Hcy in CBS-WT mouse urine was relatively high in this report, this might reflect differences in age (15–22 days in this study versus 6–9 months in the previous study) and diet.

In our previous study [10], Cys was lower in CBS-Hetero and -KO mouse plasma samples than in CBS-WT mice. In addition, CysGly in plasma samples was higher in CBS-Hetero mice but lower in CBS-KO mice. However, in the present study, Cys and CysGly in urine samples did not differ significantly among CBS-WT, -Hetero, and -KO mice. Although it was not clear why different trends were observed for plasma and urine, similar results have been reported in a study with human patients [17]. No significant difference was observed in the total GSH in urine between CBS-WT, -Hetero, and -KO mice, which was also in agreement with the mouse plasma results.

## 3. Materials and Methods

### 3.1. Chemicals and Reagents

L-Cys, D,L-Hcy, L-GSH, and CysGly were purchased from Sigma-Aldrich (St. Louis, MO, USA). Trichloroacetic acid (TCA) was obtained from Wako Pure Chemical (Osaka, Japan). Ammonium 7-fluoro-2,1,3-benzoxadiazole-4-sulfonate (SBD-F) was purchased from Dojindo (Kumamoto, Japan). TCEP was purchased from the Tokyo Chemical Industry (Tokyo, Japan). Phosphate buffered saline (PBS) was purchased from Takara Bio (Shiga, Japan). HPLC-grade acetonitrile was used. Water was purified using a Milli-Q system (Millipore, Bedford, MA, USA). All other chemicals were of analytical grade.

### 3.2. Biological Samples

Mouse urine samples for analyzing thiols in the cystathionine β-synthase (CBS) deficient homocystinuria model were obtained as follows—wild-type (CBS+/+, WT), heterozygous (CBS+/−, Hetero), and knockout (CBS−/−, KO) mice were produced by breeding CBS+/− mice (B6.129P2-Cbstm1Unc/J, Jackson Laboratory, Bar Harbor, ME, USA). All mice were bred and housed under a 12:12-h light–dark cycle (lights on at 8:00 a.m.) and with free access to food and water, at the Center of Biomedical Research, Graduate School of Medical Sciences, Kyushu University (Fukuoka, Japan). The mice were aged from 15 to 22 days. Urine samples were taken from the ureter (WT, *n* = 4; Hetero, *n* = 9; and KO, *n* = 7). All experiments were performed with the permission of the Animal Care and Use Committee of the Kyushu University (A29-037-0, March 13, 2017).

### 3.3. Sample Pretreatment

Pretreatment of mouse urine samples and standard solutions was based on methodology from a previous report [18]. In brief, the samples were incubated with tris(2-carboxyethyl)phosphine (TCEP) to reduce disulfide bonds into thiols. After deproteinization (by adding TCA) and centrifugation, biothiols were fluorescently derivatized with ammonium 7-fluoro-2,1,3-benzoxadiazole-4-sulfonate (SBD-F). The samples were diluted five times with acetonitrile. The resultant was injected into the HPLC system for analysis.

### 3.4. HPLC Conditions

The HPLC system consisted of a pump (PU-2080 Plus, JASCO, Tokyo, Japan), a column oven (CO-1560, JASCO), and a fluorescence detector (RF-20A, Shimadzu, Kyoto, Japan). An InertSustain Amide column (150 × 3.0 mm i.d., 5 µm, GL Sciences, Tokyo, Japan) was used. The mobile phase was acetonitrile-120 mM ammonium formate buffer (pH 3.0) (81:19), at a flow rate of 0.4 mL/min. The column temperature was 35 °C, and SBD-thiols fluorescence was detected, with excitation and emission wavelengths of 375 and 510 nm, respectively. The chromatograms were analyzed using the Chromato-Pro software (ver. 5.00, Run Time Corporation, Kanagawa, Japan).

The resolution was calculated from the following equation:

*R_s_* = 1.18(*t*_2_ — *t*_1_)/(*W*_0.5*h*,1_ + *W*_0.5*h*,2_), (1)

*t* is the retention time for each peak and *W*_0.5h_ is the full width at half maximum of each peak.

### 3.5. Analytical Validation

LOD and LOQ were calculated by S/N (signal to noise ratio) = 3 and 10, respectively. Quantification of SBD-thiols in mouse urine was calculated from an external standard method, without any added internal standard.

The recovery of biothiols in mouse urine samples using the developed method was examined by spiking additional thiols at three different concentrations. The recovery value was calculated as the ratio of the increase in the amount of SBD-thiols measured by the calibration curve to the amount of spiked thiol compounds.

To assess the intra- and inter-day precision, mouse urine samples at each concentration were injected in quintuplicate, on the same day and on sequential days, respectively. The precision was expressed as relative standard deviation (RSD) [%].

## Figures and Tables

**Table 1:** Limits of detection (LOD), limits of quantitation (LOQ), and linearity for SBD-thiols.

SBD-Thiols	LOD [nM]	LOQ [nM]	Linearity [nM]
	(S/N = 3)	(S/N = 10)	R^2^ > 0.999
**Hcy**	3.5	12	20–2000
**Cys**	20	67	120–12000
**CysGly**	3.9	13	30–3000
**GSH**	16	53	60–6000

**Table 2:** Intra-day precision and recovery of thiols in mouse urine samples (*n* = 5).

Thiols	Added [µM]	Measured (Mean ± SD) [µM]	RSD [%]	Recovery [%]
**Hcy**	0	13.3 ± 0.2	1.4	-
	10	23.7 ± 0.5	2.1	104
	20	34.7 ± 1.3	3.8	107
	40	54.0 ± 1.3	2.4	102
**Cys**	0	202 ± 6	3.1	-
	125	323 ± 5	1.7	97
	250	426 ± 15	3.3	104
	500	684 ± 19	2.8	96
**CysGly**	0	50.9 ± 0.5	1.1	-
	30	80.3 ± 2.1	2.6	98
	60	116 ± 4	3.1	108
	120	163 ± 3	1.8	93
**GSH**	0	7.76 ± 0.17	2.2	-
	6	13.5 ± 0.4	3.0	96
	12	22.0 ± 0.6	2.6	119
	24	34.2 ± 0.8	2.4	110

**Table 3:** Inter-day precision and recovery of thiols in mouse urine samples (*n* = 5).

Thiols	Added [µM]	Measured (Mean ± SD) [µM]	RSD [%]	Recovery [%]
**Hcy**	0	30.8 ± 1.5	4.9	-
	10	40.5 ± 1.2	2.9	97
	20	50.4 ± 2.5	5.0	98
	40	72.1 ± 2.5	3.4	103
**Cys**	0	368 ± 12	3.2	-
	125	483 ± 16	3.3	91
	250	597 ± 33	5.5	91
	500	853 ± 35	4.1	97
**CysGly**	0	116 ± 7	5.7	-
	30	143 ± 7	4.6	92
	60	171 ± 13	7.3	91
	120	233 ± 14	6.0	97
**GSH**	0	12.2 ± 0.5	3.8	-
	6	17.4 ± 0.4	2.1	87
	12	24.2 ± 1.2	4.9	100
	24	38.6 ± 1.1	2.7	110
